# EML4‐ALK‐Positive Ovarian Cancer With Intracranial Metastasis Responds to Lorlatinib: A Case Report and Literature Review

**DOI:** 10.1002/ccr3.70043

**Published:** 2025-01-07

**Authors:** Qiongqian Li, Tongze Cai, Xiaoming Zheng, Shunrong Zhang, Chanjuan Li, Huang Tang, Zhiyong Yu, Jianlong Zhou

**Affiliations:** ^1^ Department of Oncology Guangxi International Zhuang Medicine Hospital Affiliated to Guangxi University of Chinese Medicine Guangxi China

**Keywords:** EML4‐ALK fusion gene, intracranial metastasis, lorlatinib, ovarian cancer

## Abstract

We report a case showing that lorlatinib is effective in treating EML4‐ALK‐positive low‐grade serous ovarian cancer (LGSO) with intracranial metastasis. This may be the first clinical evidence of LGSO benefit from ALK inhibitors, to provide evidence for the use of ALK inhibitors in more ovarian cancer patients with EML4‐ALK fusion and promoting new ideas for the study of EML4‐ALK targets in ovarian cancer.

## 1 Introduction

Ovarian cancer is one of the three most common malignant tumors in gynecology, with the highest mortality rate; ovarian cancer has become a serious threat to women's health. Epithelial ovarian cancer is the most common type among ovarian malignancies, accounting for 80%–90%. Due to unclear early symptoms, it is mostly in the late stage when been diagnosed [1, 2]. Late‐stage ovarian cancer often loses the opportunity for radical surgery, and treatment mainly focuses on cytotoxic drugs and anti‐angiogenic targeted drugs, but it is prone to recurrence and drug resistance. In recent years, PARP inhibitors have improved the survival rate of ovarian cancer patients to some extent, but the overall 5‐year survival rate is 40%–50%, while the 5‐year survival rate of mid‐ to late‐stage ovarian cancer is less than 30% [3]. Therefore, understanding the molecular genetics of ovarian cancer and seeking treatment is necessary. Molecular targets may be a pathway to improve prognosis.

With the development of genetic testing technology, precise and personalized comprehensive treatment based on molecular level has become particularly important. Currently, research on BRCA1/2 gene mutations is the most extensive and clinically significant in ovarian cancer [4]. Targets such as HRD, BRAF, RET, HER2, and PD‐1/PD‐L1 have also emerged on the stage [5, 6], but seldom research indicates that ALK inhibitors may be effective in the treatment of EML4‐ALK+ ovarian cancer [7]. This article reports a case of EML4‐ALK‐positive advanced low‐grade serous ovarian cancer (LGSOC) with intracranial metastasis that responded dramatically to a third‐generation ALK inhibitor, lorlatinib, with good therapeutic effect. Does it indicate that EML4‐ALK may become another potential therapeutic target for ovarian cancer?

## 2 Case History and Examination

### 2.1 Medical History

A 43‐year‐old Chinese female was admitted to the Guangxi International Zhuang Medical Hospital in October 2023 with a history of headaches for 3 months. She visited a local county‐level hospital in July 2023 due to lower abdominal pain; the tumor marker test showed a significant elevation in CEA (22.58 ng/mL) and CA‐125 (62.81 U/mL). HE4 was not detected. Ultrasound showed that there was a solid mass in the left adnexal area (approximately 77 * 58 * 77 mm), suggesting ovarian cancer. On July 7, 2023, laparoscopic left adnexectomy was performed. Pathological examination showed that the fragmented tissue of the left adnexal tumor was consistent with low‐grade serous carcinoma based on morphology and immunohistochemical results: immunohistochemistry: CEA (+), CA125 (+), WT‐1 (+), CK7 (+), Pax‐8 (−), ER (−), PR (−), CD34 (−), P53 (+, wild‐type), Ki‐67 (hot spot area about 20%,+). Without receiving systematic treatment, she was discharged from the hospital.

Headache appeared one month later after the surgery. CT‐enhanced examination at another hospital showed the following results: 1. After left ovarian cancer resection, no residual lesions were found in the surgical area; 2. CT plain scan of the lungs indicates multiple bone metastases; brain MRI‐enhanced scan suggests multiple intracranial metastases. Diagnosis: 1. Ovarian cancer (low‐grade serous carcinoma, IVB stage/FIGO 2018); 1.1. Intracranial metastasis; 1.2. Bone metastasis.

From September 2023 to February 2024, the patient underwent regular first‐line treatment with paclitaxel+carboplatin+bevacizumab regimen for 3 cycles, and response evaluation is PD according to RECIST1.1; then, the patient underwent second‐line treatment with gemcitabine+carboplatin+bevacizumab for 3 cycles and whole brain radiation therapy (30Gy/15Fx). During the treatment process, she suffered severe bone marrow suppression (thrombocytopenia grade IV), worsening headache symptoms, and significantly increasing CEA, HE4, and CA125. What's more, CT showed an increase in bone lesions compared to before. The response evaluation was PD.

### 2.2 Methods

**FIGURE 1:**
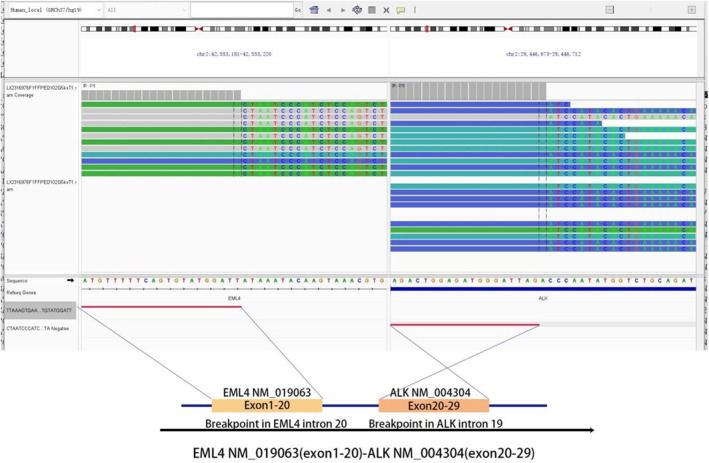
NGS assay detected that exons 1–20 of EML4 were fused to exons 20–29 of anaplastic lymphoma kinase(ALK) through intron20 of EML4 and intron29 of ALK. Visualized in the integrative genomics viewer.

Blood sample NGS detection report (solid tumor 102 gene): EML4‐AKL exon20 mutation abundance is 16.81%, and other commonly used genes such as BRCA, BRAF, NTRK, RET, and PD‐L1 are not mutated (Figure 1).

## 3 Conclusion and Results

**FIGURE 2:**
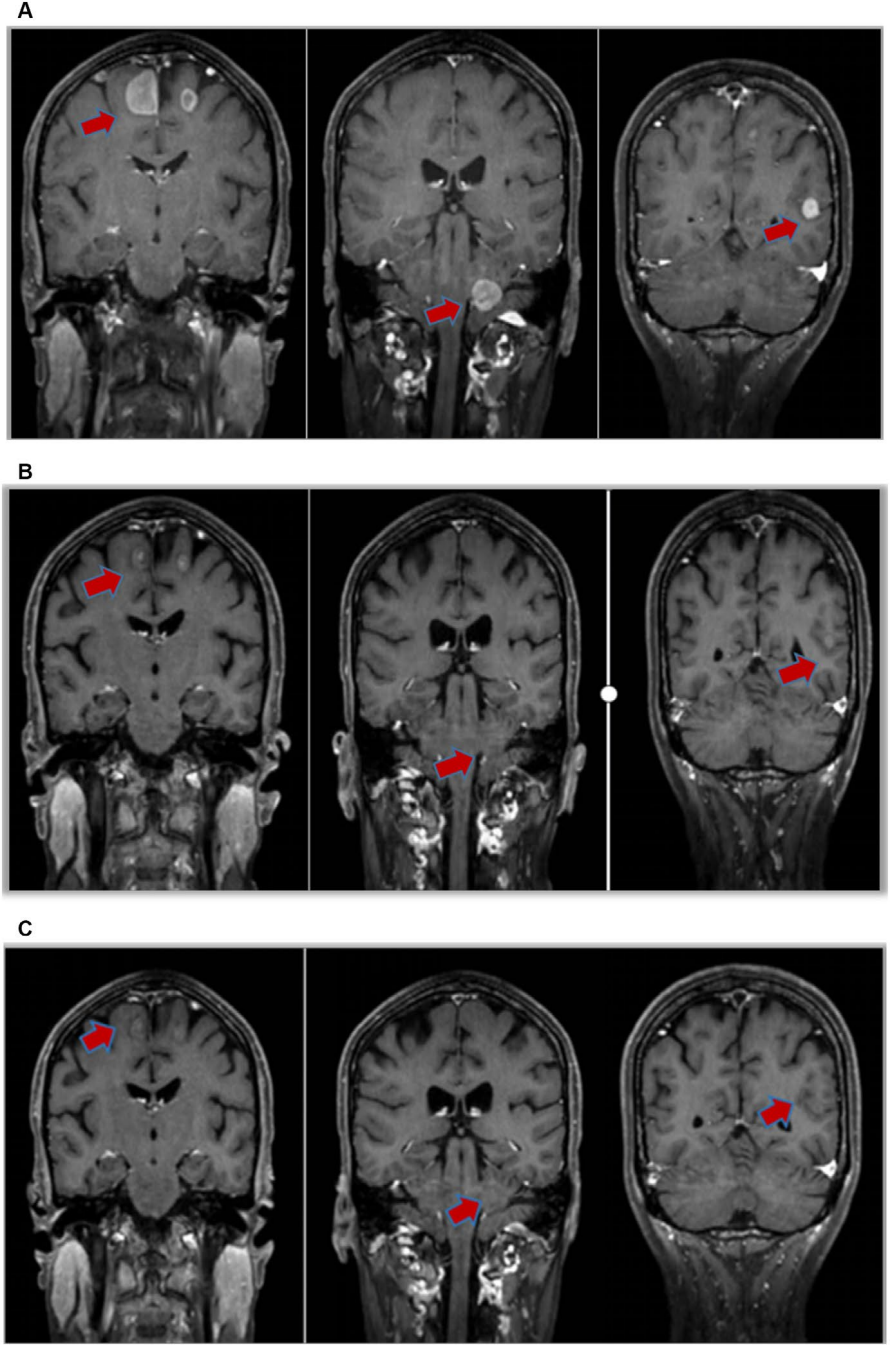
(A) Basal line. (B) One month after taking lorlatinib. Clinical responses to lorlatinib. The patient achieved a partial response (PR) after 1 month of administration of lorlatinib. (C) Two months after taking lorlatinib, almost all of the intracranial metastasis lesions disappeared.

**FIGURE 3:**
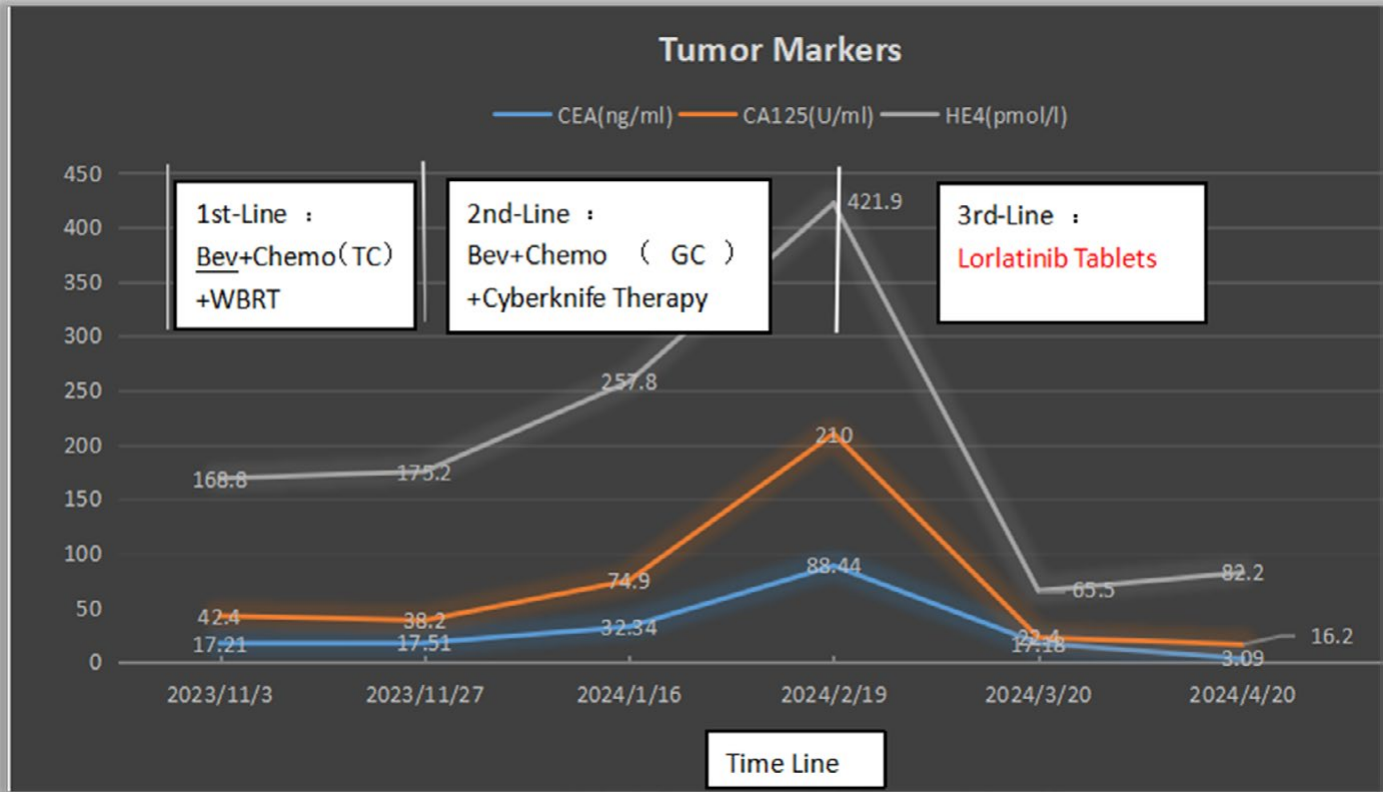
The patient's tumor markers level from the time of first‐line treatment to the last follow‐up of lorlatinib treatment. Bev, bevacizumab; GC, gemcitabine+carboplatin; TC, albumin paclitaxel+carboplatin; WBRT, whole brain radiotherapy treatment.

At present, there is no relevant clinical research or indication for the usage of ALK inhibitors in the field of ovarian cancer treatment. However according to clinical research on ALK‐positive lung cancer, ALK inhibitors have been proven effective. After consultation, the patient took lorlatinib tablets 100 mg for targeted treatment of her own accord on February 21, 2024, and regular denosumab injection for bone improvement treatment. In March 2024, a follow‐up examination showed that the intracranial metastasis lesion had shrunk, the bone lesion was stable, tumor markers had decreased, and headache symptoms had significantly improved. Treatment benefits are considered (PR according to RECIST1.1). Brain MRI showed continuous shrinkage of intracranial metastases in April 2024. The comparison of imaging/tumor markers before and after treatment is shown below (Figures 2 and 3).

## 4 Discussion

Epithelial ovarian cancer (EOC) has a hidden onset, with about 70% of patients diagnosed as advanced stage. Surgery, chemotherapy, and targeted therapy are the main treatment methods, but the long‐term survival rate of patients has not significantly improved, and the mortality rate ranks first among malignant tumors in the female reproductive system [8]. Since 1996, based on three independent randomized trials (GOG111, OV10, and GOG 258), the combination of platinum drugs and paclitaxel has been established as the first‐line standard treatment for advanced EOC [9, 10]. In 2011, according to the data from phase III GOG‐218 and ICON‐7 clinical trials, NCCN guidelines prioritized the use of bevacizumab‐containing regimens for first‐line treatment of stage II‐IV ovarian cancer patients [11, 12]. In this case, the patient did not benefit from this treatment plan. During the treatment process, tumor markers CEA, CA125, and HE4 increased, and headache worsened indicating a poor prognosis [13, 14, 15]. Therefore, it is necessary to understand the overall genetic information of the patient and strive to find breakthrough points in treatment.

**FIGURE 4:**
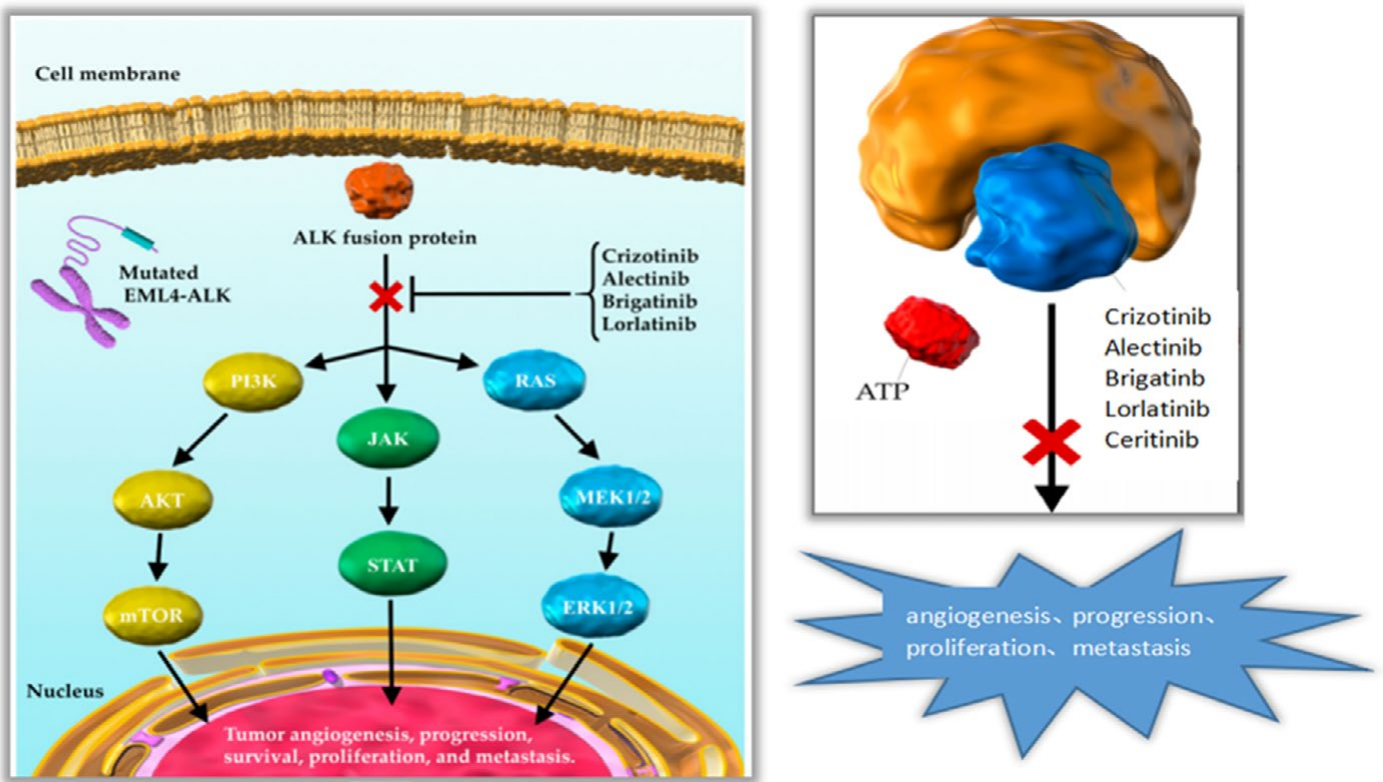
Formation of EML4‐ALK4 fusions, activation of downstream pathways, and how ALK‐TKIS works.

Anaplastic lymphoma kinase (ALK) was first discovered in the subtype of anaplastic large‐cell lymphoma in 1994, and genetic abnormalities may be closely related to the development, progression, and prognosis of various solid tumors [16, 17]. Soda et al. found for the first time that both ALK and EML4 genes are located on the same short arm of human chromosome 2, but in opposite directions, small inversions involving both loci lead to gene fusion [18]. The fusion gene EML4‐ALK and its activated tyrosine kinase function can induce downstream signaling pathways and promote cell proliferation and survival as shown in Figure 4.

ALK has been extensively studied in non‐small cell lung cancer, and the FDA has also approved five ALK inhibitors—alectinib, bugatinib, seretinib, clozotinib, and lorlatinib—for non‐small cell lung cancer with abnormal ALK expression [19, 20]. In recent years, there have been reports of abnormal ALK expression in ovarian cancer [21, 22, 23, 24], However, there is only one case report of ALK inhibitors being used in high‐grade serous ovarian cancer [7] without intracranial metastasis; in this case, the patient suffers from low‐grade serous ovarian cancer with intracranial metastasis; we chose lorlatinib tablets with better blood–brain barrier effect and lower resistance mutation rate (Table 1), which achieved good efficiency. This will become the first clinical evidence suggesting that ALK inhibitors may be an effective treatment for EML4‐ALK+ low‐grade serous ovarian cancer with intracranial metastasis.

**TABLE 1:** Approval of ALK inhibitors and blood–brain barrier penetration.

Drugs	FDA approved indication [25]	Blood–brain barrier penetration [26, 27]	Intracranial ORR [28]	BM (NSCLC) PFS [29]	IC50 [25]
Crizotinib	Unresectable, recurrent, or refractory ALK‐positive IMT Unresectable, recurrent, or refractory ALK‐positive ALCL	Low	0.252	0.392	24 nm
	ALK‐positive unresectable/metastatic NSCLC who had previously received one platinum‐containing regimen				
Ceritinib	Unresectable or metastatic ALK‐positive NSCLC after progression or intolerance to crizotinib	higher	0.457	0.395	0.2 nm
Brigatinib	Unresectable or metastatic ALK‐positive NSCLC	higher	0.671	0.727	0.37 nm
Alectinib	Unresectable or metastatic ALK‐positive NSCLC	higher	0.727	0.775	1.9 nm
Lorlatinib	Unresectable or metastatic ALK‐positive NSCLC	higher	0.787	0.973	0.07 nm

Perhaps this case has its specificity, but with the increasing number of ovarian cancer patients and the advancement and development of genetic testing technology, it is hoped that the report of this case can promote the search for more suitable targeted therapy groups, provide evidence for the use of targeted drugs in more ovarian cancer patients with EML4‐ALK fusion, and promote new ideas for the research of EML4‐ALK targets in ovarian cancer.

## Author Contributions

**Jianlong Zhou** and **Zhiyong Yu** came up with the clinical treatment strategies. **Qiongqian Li:** doctor in charge; manuscript writing. **Tongze Cai:** literature search; revision; **Xiaoming Zheng** and **Shunrong Zhang** created figures and tables; **Huang Tang** and **Chanjuan Li** polished a thesis. All authors discussed the results and contributed to the final manuscript.

## Ethics Statement

The authors have nothing to report.

## Consent

Written informed consent was obtained from the patient to publish this report by the journal's patient consent policy.

## Conflicts of Interest

The authors declare no conflicts of interest.

## Data Availability

Data is openly available in a public repository that issues datasets with DOIs.
